# On the Physiology of the Division of Tendons in Surgical Operations, Illustrated by Experiments

**Published:** 1838-04

**Authors:** 


Art. VIII.—*De Physiologia Tenotomiæ Experimentis illustrata. Commentatio Chirurgica, qua Ordini Medicorum gratioso Acad. Georg. Aug. &c. &c. gratulatur* Fred. Aug. ab Ammon, m.d., Reg. Saxon. Archiater, &c. &c.—*Dresdæ*, 1837. 4to. pp. 24.

*On the Physiology of the Division of Tendons in Surgical Operations, illustrated by Experiments.* By F. A. von Ammon, m.d., Chief Physician to the King of Saxony.—*Dresden*, 1837. 4to. pp. 24; *with a Plate.*

This short treatise is a gratulatory address presented to the university of Göttingen, the 17th September, 1837, on the centenary of its institution. The distinguished author justly considers that the investigation of this subject is one of much interest and importance at the present time, more especially as the particular mode of practice referred to must still be regarded as in its infancy, and has hitherto been very imperfectly examined in a physiological point of view. His claim to the right of discussing it is founded on his having repeatedly performed the operation on man, and, in an experimental way, on animals.

After some introductory matter respecting the nomenclature and synonymy, &c., the author proceeds to give the history of the operation. Like many other medicinal and chirurgical modes of treatment, the operation for the division of tendons, or, as the author, following some French writers, terms it, *Tenotomia*, or *Tenotomy*, (from the Greek words τενων and τομη,) has, since its first introduction, undergone many vicissitudes of fame and favour; falling for a time into discredit and disuse, and then being restored with all the pomp of a new discovery. According to Von Ammon, the operation appears to have been first performed (on the tendon of the mastoid muscle) by Roger Roonhuysen, in the latter part of the seventeenth century. He was followed by Meekren, Tulp, Blasius, Ten Haaf, and, much more recently, by Thilenius, Michaelis, and Sartorius: the two latter of whom divided the tendo Achillis for the cure of clubfoot. Some fifteen years afterwards, Delpech taught and practised the operation; and subsequently Dupuy adopted it, but professedly as the imitator of the Dutch surgeons, not of his own countrymen. Lafosse, in France, introduced the operation into veterinary practice about the end of the last century; and it seems to have been subsequently followed in that country, with good success, in the cure of contractions of the tendons in the feet of horses. Ten years after Delpech, Stromeyer, of Hanover, once more set about curing clubfoot by the division of the tendo Achillis, and undertook a set of experiments on the result of similar operations on animals, in conjunction with Dr. Gunther, veterinary professor in the same city. In consequence of this new impulse given to the practice, it was speedily followed by Dieffenbach, Holscher, Elster, Bünger, Leonhard, Zeis, Ulrich, Von Ammon, &c. in Germany; by Bouvier, Roux, Cazenave, &c. in France; and, we may add, by Dr. Little, Mr. Whipple, and many other surgeons, in England. The general result of these operations has been most satisfactory; and the operation may now be considered as too well established to be again allowed to fall into disuse.

The author attributes the slow and uncertain progress of the operation of *tenotomy* to various circumstances; to the general opinion of the difficulty with which tendons were healed when ruptured; the apprehension of bad consequences, such as tetanus and spasm from incised wounds of them; prejudices probably derived from the confounding by the ancients, both in name and in fact, of the nerves with the tendons. It was only by the results of actual practice, and by the experimental investigation of the whole subject, that these false notions and fears could be dissipated.

The experiments made of late years by Duval have thrown much light on the natural processes in cases of this kind; and it is but justice to this author to state that the conclusions published by him coincide perfectly with those deduced by Professor von Ammon from his experiments.

These, in which he was assisted by Dr. Prinz, the veterinary professor, and by Dr. Zeis, of Dresden, were performed on six horses and three rabbits. In the horses, the tendon of the flexor profundus of one of the fore feet, and in the rabbits the tendo Achillis, was divided: the effects resulting from the wounds being carefully observed, the animals were killed at different periods after the operation, and the state of the wounded parts closely examined. The local reaction and inflammatory fever varied much in the different cases. The following are the general results observed on dissection:—1. Twenty-four hours after the operation, the whole wound was found filled with coagulated blood, or there was a coagulum on the exremities of the divided tendon, without any traces of plastic or coagulable lymph, or of new vessels. 2. In one instance, in the horse, some traces of plastic lymph were found on the second day. 3. After the fourth day, the lymph presented small, conical elevations on the cut ends of the tendon, (which were still surrounded with coagulated blood,) so as partially to lessen the distance between the divided parts. 4. After the seventh day, these elevations assumed the appearance of threadlike elongations uniting the divided tendon, and the coagulum appeared intermixed with plastic lymph. 5. After a month the wound was perfectly cicatrized, and the new production resembled tendon in its structure, although still distinguishable from the original structure at the point of union: there was no inflammation in the neighbouring parts. 6. After the thirty-sixth day (the longest period of observation), the new substance, in the horse's foot, had more resemblance to cellular tissue, but was firm, of a blueish-red colour; the tendon was somewhat elongated. All the foregoing results were obtained on the horse. In two of the rabbits, examined after six days, the results are not clearly detailed; in the third, the tendon, after the twelfth day, was found united by a slender, hard, and very compact substance, almost like the original tissue, and adherent to the neighbouring parts. The author regards the new-formed substance not as actual tendon, being distinguished from it by its inferior smoothness and lustre and greater density, as well as by its colour, which at first is reddish, and subsequently blueish; but considers it as having precisely the same function as the primary tendinous tissue. He believes that the new substance requires about fourteen days for its formation. It is important to add, that Dr. Hartwig, who gives a brief analysis of Professor von Ammon's dissertation in the *Medicinische Zeitung* for January 17th, informs us that he has performed the same experiment on the ligamentum nuchæ, and flexor tendon of the foot in upwards of twenty horses, and, for the most part, with very similar results. We regard the profession as much indebted to Professor von Ammon for this short treatise. Although it contains nothing very new, it places the facts of the case in a clear light, and gives a more connected view of the history and whole bearings of the operation than we before possessed.

